# *Staphylococcus aureus* Bacteremia, Europe

**DOI:** 10.3201/eid1111.050524

**Published:** 2005-11

**Authors:** Edine W. Tiemersma, Dominique L. Monnet, Nienke Bruinsma, Robert Skov, Jos C.M. Monen, Hajo Grundmann, European Antimicrobial Resistance Surveillance System participants

**Affiliations:** * National Institute for Public Health and the Environment, Bilthoven, the Netherlands; † Statens Serum Institut, Copenhagen, Denmark

**Keywords:** Staphylococcus aureus, surveillance, bacteremia, letter

**To the Editor:** In their article, Collignon et al. (*1*) present a table comparing absolute numbers and incidence rates of *Staphylococcus aureus* bacteremia (SAB) in Australia to those of 5 other countries, and state that "some data are available from other countries for comparison" and "only 2 countries, Denmark and England, appeared to have comprehensive collection systems."

We would like to add data from the European Antimicrobial Resistance Surveillance System (EARSS) to their table. EARSS is a multinational surveillance system that links national networks by collecting comparable and validated data on the prevalence of antimicrobial resistance of 5 microorganisms, including *S. aureus* (*2*). A total of 30 countries participate in EARSS. To ensure representativeness of the data it publishes, EARSS has set criteria, which can be found elsewhere (*3*).

In 2003, through a questionnaire, we collected background information on all hospitals served by laboratories participating in EARSS, including the estimated hospitals' catchment populations. Proportion of the country population covered by EARSS was then calculated by dividing the sum of catchment populations by the total population of the country (*4*). Catchment populations of hospitals providing single specialty or supra-regional type of care were not counted to avoid overlap with other hospitals within the same country. Only the countries that provided denominator data for at least 60% of the isolates were included to ensure that the sample of hospitals was still representative of the country as a whole.

The number of SAB and the incidence of SAB per 100,000 inhabitants in 2003 were calculated from EARSS data, adjusted for population coverage, and are presented in the Table. These are crude estimates of the true number of SAB and should, thus, be interpreted with caution. For example, we assumed that the isolates for which hospital background information was not available did not differ from isolates for which we had this information, and that hospitals that participated in EARSS in 2003 were a representative sample of the countries' hospitals. Additionally, the incidence of SAB was positively correlated with the blood culturing rate (Spearman *r* = 0.74, p = 0.002), which means that the incidence of SAB is likely to have been underestimated in countries that reported few blood cultures. Although some countries did not report their blood culturing rate, the incidences of SAB in these countries were among the highest and are unlikely to be underestimated. For example, the EARSS data for Denmark and Ireland nicely fitted those presented in Table 4 of the article by Collignon et al. (*1*). Finally, reporting to the EARSS system greatly improved over the years, which is why this study was performed on the last available year, 2003. Nevertheless, one cannot exclude underreporting of SAB by EARSS participating hospitals since EARSS is a voluntary reporting system. For example, England reported 18,403 SAB cases or an incidence of 37 SAB per 100,000 inhabitants from April 2002 to March 2003 through its mandatory surveillance scheme (*5*), whereas an estimate for the United Kingdom from the EARSS database would only give 7,800 SAB cases for 2003. However, it is impossible to determine whether this discrepancy was due to poor voluntary reporting of SAB cases, a lower blood culturing rate in EARSS participating hospitals, or a poorly representative sample of the country's hospitals. Data from the United Kingdom were excluded from the present study on the basis of the latter possibility; denominator information for <60% of the isolates was available.

**Table:** Absolute numbers, rates of Staphylococcus aureus bacteremia (SAB), and percentage of methicillin-resistant Staphylococcus aureus (MRSA), European Antimicrobial Resistance Surveillance System (EARSS), 2003*

Country	Population† (4)	% population covered by EARSS‡	Blood culture sets/1,000 inhabitants	No. SAB reported to EARSS	No. SAB for country§	SAB/100,000 inhabitants§	% MRSA
Austria	8,188,207	42.7	NA	871	2,038	25	15
Bulgaria	7,537,929	100	2	157	149	2¶	31
Croatia	4,422,248	81.3	7	360	443	10	37
Czech Republic	10,249,216	92.3	11	1,387	1,503	15	7
Denmark	5,384,384	46.2	NA	671	1,451	27	<1
Estonia	1,408,556	100	<1	98	98	7¶	5
Finland	5,190,785	94.3	27	727	771	15	1
Hungary	10,045,407	100	1	859	859	9¶	14
Iceland	280,798	100	28	64	64	23	0
Ireland	3,924,140	89.2	NA	1,108	1,243	32	42
Israel	6,116,533	39.7	42	368	926	15	43
Malta	400,420	100	4	122	122	31	43
Poland	38,622,660	24.3	3	166	684	2¶	19
Romania	22,271,839	59	<1	85	144	<1¶	46
Slovenia	1,935,677	100	17	299	296	15	13
Spain	40,217,413	24.3	21	1,391	5,731	14	25
Sweden	8,878,085	100	28	1,855	1,760	20	<1

*Only countries that provided hospital background information for at least 60% of the isolates were included; NA, not available. †Source (*4*). ‡Population coverage rate as calculated from EARSS hospitals that provided background information was adjusted for nonresponding hospitals as follows: population coverage as calculated divided by proportion of isolates with hospital background information. §The total number of SAB per country was calculated as follows: number of *S. aureus* isolates in EARSS divided by adjusted proportion of population covered. ¶These rates are grossly underestimated because of the very low blood culturing rate.

In conclusion, EARSS is the first comprehensive surveillance system on antimicrobial resistance in Europe. Within certain limitations, EARSS can also provide valuable information on blood-culturing practices and the incidence of SAB in Europe. The system is continuously being improved, and additional information on the representativeness of EARSS data is being collected. This will allow us to improve the quality and accuracy of the reported incidence rates. In the future, the system should allow reporting of similar data for an even larger number of European countries and for additional microorganisms, such as *Escherichia coli*.
